# Comprehensive Analysis of Hexokinase 2 Immune Infiltrates and m6A Related Genes in Human Esophageal Carcinoma

**DOI:** 10.3389/fcell.2021.715883

**Published:** 2021-10-07

**Authors:** Xu-Sheng Liu, Jia-Min Liu, Yi-Jia Chen, Fu-Yan Li, Rui-Min Wu, Fan Tan, Dao-Bing Zeng, Wei Li, Hong Zhou, Yan Gao, Zhi-Jun Pei

**Affiliations:** ^1^Department of Nuclear Medicine and Institute of Anesthesiology and Pain, Taihe Hospital, Hubei University of Medicine, Shiyan, China; ^2^Hubei Clinical Research Center for Precise Diagnosis and Treatment of Liver Cancer, Taihe Hospital, Hubei University of Medicine, Shiyan, China; ^3^Shiyan Emergency Medical Center, Shiyan, China; ^4^School of Public Health, Hubei University of Medicine, Shiyan, China

**Keywords:** HK2, esophageal carcinoma, immune infiltration, m6A modification, tumor microenvironment

## Abstract

**Background:** Hexokinase 2 not only plays a role in physiological function of human normal tissues and organs, but also plays a vital role in the process of glycolysis of tumor cells. However, there are few comprehensive studies on HK2 in esophageal carcinoma (ESCA) needs further study.

**Methods:** Oncomine, Tumor Immune Estimation Resource (TIMER), The Cancer Genome Atlas (TCGA) and Gene Expression Omnibus (GEO) database were used to analyze the expression differences of HK2 in Pan-cancer and ESCA cohort, and to analyze the correlation between HK2 expression level and clinicopathological features of TCGA ESCA samples. GO/KEGG, GGI, and PPI analysis of HK2 was performed using R software, LinkedOmics, GeneMANIA and STRING online tools. The correlation between HK2 and ESCA immune infiltration was analyzed TIMER and TCGA ESCA cohort. The correlation between HK2 expression level and m6A modification of ESCA was analyzed by utilizing TCGA ESCA cohort.

**Results:** HK2 is highly expressed in a variety of tumors, and its high expression level in ESCA is closely related to the weight, cancer stages, tumor histology and tumor grade of ESCA. The analysis results of GO/KEGG showed that HK2 was closely related to cell adhesion molecule binding, cell-cell junction, ameboidal-type cell migration, insulin signaling pathway, hif-1 signaling pathway, and insulin resistance. GGI showed that HK2 associated genes were mainly involved in the glycolytic pathway. PPI showed that HK2 was closely related to HK1, GPI, and HK3, all of which played an important role in tumor proliferation. The analysis results of TIMER and TCGA ESCA cohort indicated that the HK2 expression level was related to the infiltration of various immune cells. TCGA ESCA cohort analyze indicated that the HK2 expression level was correlated with m6A modification genes.

**Conclusion:** HK2 is associated with tumor immune infiltration and m6A modification of ESCA, and can be used as a potential biological target for diagnosis and therapy of ESCA.

## Introduction

Recent studies show that Esophageal carcinoma (ESCA) ranks seventh in terms of incidence and sixth in mortality overall (Sung et al., 2021). Despite substantial improvements in the diagnosis and treatment of esophageal diseases, the prognosis for patients with ESCA remains poor (Bray et al., 2018). The occurrence and development of ESCA is an extremely complex biological process, and the expression of many genes has changed. Therefore, further research on the molecular mechanism of ESCA can provide new theoretical value for the diagnosis and treatment of tumors.

Hexokinase (HK) is an enzyme capable of phosphorylating hexose and is a rate-limiting enzyme in the glycolytic pathway. Four subtypes of human HK have been discovered, which are encoded by the genes HK1, HK2, HK3, and HK4, respectively (Zhang et al., 2017). HK1 is widely expressed in mammalian tissues, HK2 is usually expressed in insulin-sensitive tissues such as fat, bone and heart muscle, and HK3 is expressed at a low level, while HK4 expression is limited to pancreas and liver (Smith, 2000; Zhang et al., 2017). HK2 not only plays a role in physiological function of human normal tissues and organs, but also plays an important role in the glycolysis of tumor cells (Yu et al., 2021). Currently, researchers have found that expression of HK2 is increased in a variety of tumors and promotes the development of tumors (Shen et al., 2020; Wang et al., 2020). Our previous study found that HK2 was highly expressed in ESCA, but no more studies were conducted on the biological function of HK2 (Liu et al., 2020b).

Tumor immunotherapy and N6-methyladenosine (m6A) modification are hot spots in tumor treatment, which are extensively used in the investigation and therapy of ESCA (Baba et al., 2020; Wu S. et al., 2020; Wu X. et al., 2020; Yang et al., 2020). However, there are less investigates on the overall understanding of HK2 in ESCA, particularly the correlation between HK2 and ESCA immune cell infiltration and m6A modification.

On this project, we processed The Cancer Genome Atlas (TCGA) ESCA cohort and performed bioinformatics analysis using the R software, online website and other databases. The differences of HK2 expression in different cancer tissues were studied, and the mRNA and protein expression of HK2 in ESCA were verified by cell assay and immunohistochemistry (IHC). The co-expression gene networks of HK2 in ESCA were analyzed, and the possible biological mechanisms and signal pathways involved in associated genes were analyzed. Eventually, the correlation between the expression difference of HK2 and the tumor immune cell infiltration and m6A modification of ESCA will be explored, which will help to study the potential pathogenesis of ESCA.

## Materials and Methods

### Ethics Statement

The protocol of this study had been approved by the Ethics Committee of Taihe Hospital Affiliated of Hubei University of Medicine (Shiyan, China) (document NO.2021KS021) and conducted according to the principles stated in the Declaration of Helsinki, and the requirement to obtain informed consent was waived.

### Expression of Hexokinase 2 in Pan-Cancer and Esophageal Carcinoma

Oncomine^1^ (Rhodes et al., 2004, 2007) and TIMER^2^ (Li et al., 2016, 2017) databases were used to analyze the expression level of HK2 in different tumors. Oncomine database used Student’s *t*-test to compare the transcription level of HK2 in clinical cancer samples and normal control group, and selected data with multiple change > 2 and *P*-value < 0.01. We also downloaded the RNA sequencing data of ESCA from TCGA^3^ (Tomczak et al., 2015) and GEO (GSE38129)^4^ (*n* = 60; GSE23400, *n* = 106) cohort to analyze the difference of HK2 expression between ESCA and normal tissues. UALCAN database^5^ (Chandrashekar et al., 2017) was used to analyze the relationship between the expression level of HK2 and the clinicopathological characteristics of ESCA patients. Eventually, we validated the mRNA and protein expression of HK2 in ESCA and control sample by qRT-PCR and IHC assay according to the method described previously (Liu et al., 2020a, 2021a), as detailed in Supplementary Materials.

### Enrichment Analysis of Hexokinase 2 Gene Co-expression Network

The co-expression network of HK2 in TCGA ESCA cohort was analyzed using LinkedOmics^6^ database. Pearson correlation coefficient was used for statistical analysis, and volcanic maps and heat maps were used for display. The rank criterion was an FDR < 0.05. The ClusterProfiler software package of R was used to analyze the Gene Ontology (GO) function and Kyoto Encyclopedia of Genes and Genomes (KEGG) pathway that co-expressed genes may participate in, and the ggplot2 software package was used to visually analyze the data.

### Analysis of Gene-Gene Interaction and Protein-Protein Interaction of Hexokinase 2 Gene

We use GeneMANIA database^7^ (Warde-Farley et al., 2010) to query and generate a list of genes that have similar functions with the HK2 gene and constructed an interactive network to illustrate the relationship between genes. The PPI of HK2 protein was calculated and predicted by using the STRING database^8^ (Szklarczyk et al., 2019).

### Correlation Analysis of Hexokinase 2 and Immune Infiltrating Cells

The correlation between HK2 and immune infiltrating cells in ESCA samples was evaluated using the TIMER database. Immune infiltrating cells include B cell, neutrophil, CD4^+^ T cell, macrophage, CD8^+^ T cell, and dendritic cell. The somatic copy number alteration (SCNA) module of the TIMER tool was used to associate the genetic copy number variation (CNV) of HK2 with the relative abundance of tumor infiltrating cells. The CIBERSORT (Newman et al., 2015) software package of R was utilized to analyze the differences of 22 immune cells between high and low HK2 expression groups in ESCA samples. In addition, we also analyzed the correlation between HK2 and immune cell markers in ESCA samples utilizing TIMER databases and TCGA ESCA cohort. Immunofiltration marker genes refer to the previous study (Liu et al., 2021b).

### Correlations of Hexokinase 2 Expression With m6A Associated Genes in Esophageal Carcinoma

The R software package was utilized to evaluate the correlation between the expression of HK2 and the expression of m6A associated gene in the TCGA ESCA cohort, including ZC3H13, YTHDF3, HNRNPC, METTL14, HNRNPA2B1, IGF2BP1, METTL3, WTAP, RBM15, ALKBH5, IGF2BP2, RBMX, RBM15B, YTHDC1, VIRMA, IGF2BP3, YTHDC2, YTHDF1, FTO, and YTHDF2 (Li et al., 2019). The R software was utilized to analyze the differences of 20 m6A associated genes between high and low HK2 expression groups in ESCA samples. The data were analyzed visually by ggplot2 software package.

## Results

### Transcriptional Levels of Hexokinase 2 in Pan-Cancer

**FIGURE 1:**
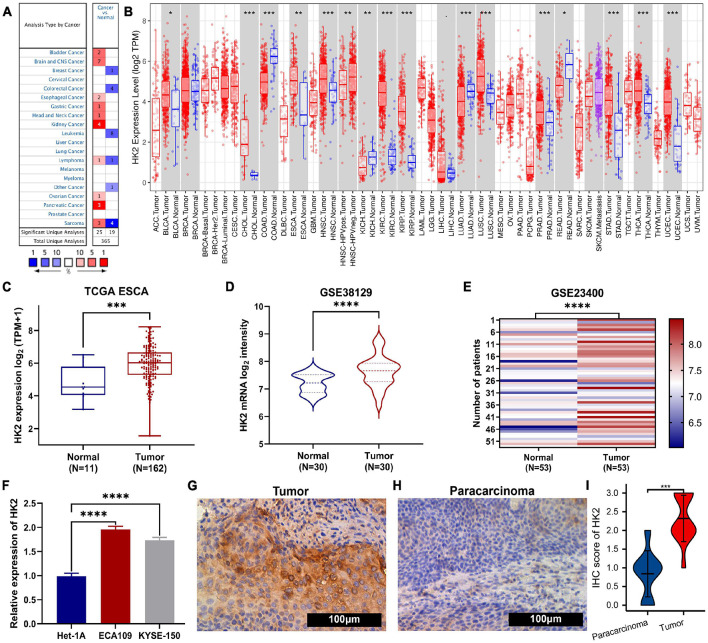
The expression of HK2 in esophageal carcinoma (ESCA) and pan-carcinoma. **(A)** Oncomine analysis showed the expression level of HK2 mRNA in different tumor types. **(B)** The TIMER ESCA cohort shows that HK2 mRNA expression levels in different tumor types. **(C)** Differential expression of HK2 in ESCA and normal tissues was analyzed by TCGA ESCA cohort **(D)** Difference expression of HK2 in ESCA and normal tissues was analyzed by GSE38129 cohort. **(E)** Difference expression of HK2 between ESCA and matched normal tissues in GSE23400 cohort. **(F)** Difference expression of HK2 in different ESCA cell lines and human normal epithelial cell lines. Immunohistochemical staining showed the expression of HK2 in ESCA samples **(G)** and paracarcinoma samples **(H)**. **(I)** The mean HK2 IHC score in ESCA tissue (2.32 ± 0.621) was significantly higher than that of matched peritumoral tissue (0.84 ± 0.618). ^∗^*P* < 0.05; ^∗∗^*P* < 0.01; ^∗∗∗^*P* < 0.001; ^*⁣*⁣**^P < 0.0001.

We analyzed the difference of HK2 mRNA expression between ESCA samples and normal tissue samples using Oncomine and TIMER databases, respectively. Oncomine database analysis showed that HK2 expression in bladder (Sanchez-Carbayo et al., 2006), brain and CNS (Bredel et al., 2005; French et al., 2005; Murat et al., 2008; Sun et al., 2006), esophageal (Hao et al., 2006), gastric (D’Errico et al., 2009), head and neck (Vasko et al., 2007), kidney (Jones et al., 2005; Gumz et al., 2007; Beroukhim et al., 2009), ovarian (Yoshihara et al., 2009), and pancreatic adenocarcinoma (Iacobuzio-Donahue et al., 2003; Badea et al., 2008; Pei et al., 2009) was higher than that in normal tissue samples. However, the expression of HK2 in breast cancer (Curtis et al., 2012), colorectal cancer (Kaiser et al., 2007; Gaedcke et al., 2010; Hong et al., 2010) and leukemia (Stegmaier et al., 2004; Andersson et al., 2007; Haferlach et al., 2010) was lower than that in normal tissues. Confusingly, there are different data suggesting that HK2 may be highly expressed in both lymphoma (Piccaluga et al., 2007) and normal tissue samples (Storz et al., 2003), and it is speculated that this may be due to different sample sizes (Figure 1A). HK2 may be highly expressed in both sarcomas (Detwiller et al., 2005) and normal tissue samples (Barretina et al., 2010), presumably due to the difference in tissue origin of the tissues designated as normal. Table 1 provides the corresponding summary results in detail.

**TABLE 1:** HK2 expression in cancerous vs. normal tissue in ONCOMINE.

**Cancer Site**	**Cancer type**	***P*-value**	***t*-test**	**Fold change**	**References (PMID)**
Bladder	Infiltrating bladder urothelial carcinoma	1.47E-12	8.112	3.119	16432078
	Superficial bladder cancer	1.51E-16	10.627	4.207	16432078
Brain and CNS	Anaplastic oligoastrocytoma	0.001	5.698	4.095	16357140
	Brain glioblastoma	1.85E-8	14.506	5.631	TCGA Brain
	Glioblastoma	0.006	3.978	5.856	TCGA Brain
	Glioblastoma	8.11E-5	6.873	3.455	16204036
	Oligodendroglioma	1.50E-6	5.268	2.460	16616334
	Anaplastic astrocytoma	8.43E-5	4.200	2.491	16616334
	Glioblastoma	1.41E-4	7.815	2.820	18565887
Breast	Breast phyllodes tumor	3.98E-4	–6.710	–2.365	22522925
Colorectal	Cecum adenocarcinoma	1.20E-6	–6.579	–2.080	17615082
	Rectal adenocarcinoma	4.02E-17	–9.720	–2.146	20725992
	Rectal mucinous adenocarcinoma	1.33E-4	–4.615	–2.337	TCGA Colorectal
	Colorectal carcinoma	1.05E-5	–5.676	–2.198	20143136
Esophageal	Barrett’s esophagus	0.003	2.929	3.157	16952561
	Esophageal adenocarcinoma	9.76E-4	3.433	3.062	16952561
Gastric	Gastric mixed adenocarcinoma	3.09E-5	5.339	2.638	19081245
Head-Neck	Thyroid gland papillary carcinoma	2.29E-4	4.668	2.487	17296934
Kidney	Hereditary clear cell renal cell Carcinoma	7.96E-29	30.305	13.568	19470766
	Non-hereditary clear cell renal cell Carcinoma	4.45E-18	16.554	8.081	19470766
	Clear cell renal cell carcinoma	1.19E-7	10.778	14.045	17699851
	Clear cell renal cell carcinoma	5.80E-10	9.709	4.871	16115910
Leukemia	Acute myeloid leukemia	4.30E-5	–5.734	–3.633	14770183
	B-Cell acute lymphoblastic leukemia	1.74E-10	–15.717	–7.694	17410184
	Acute myeloid leukemia	5.30E-7	–7.117	–2.898	17410184
	T-Cell acute lymphoblastic leukemia	1.94E-6	–7.774	–9.190	17410184
	B-Cell acute lymphoblastic leukemia	2.46E-30	–13.938	–2.545	20406941
	Chronic Lymphocytic Leukemia	2.19E-38	–20.361	–3.118	20406941
	T-Cell acute lymphoblastic leukemia	1.77E-25	–12.090	–2.282	20406941
	B-Cell childhood acute lymphoblastic Leukemia	1.07E-26	–13.463	–2.221	20406941
Lymphoma	Unspecified peripheral T-cell Lymphoma	2.43E-10	7.895	2.280	17304354
	Cutaneous follicular lymphoma	1.68E-4	–4.963	–2.329	12713594
Ovarian	Ovarian serous adenocarcinoma	1.00E-4	5.492	9.472	19486012
Pancreas	Pancreatic carcinoma	9.31E-10	8.709	6.691	19732725
	Pancreatic ductal adenocarcinoma	1.92E-11	7.825	3.038	19260470
	Pancreatic adenocarcinoma	0.005	3.955	8.921	12651607
Sarcoma	Synovial sarcoma	2.43E-5	5.554	3.867	15994966
	Round cell liposarcoma	1.07E-4	5.031	3.686	15994966
	Pleomorphic liposarcoma	5.83E-4	4.806	5.595	15994966
	Leiomyosarcoma	2.03E-10	–11.795	–5.138	20601955
	Dedifferentiated liposarcoma	3.12E-9	–10.393	–4.032	20601955
	Myxofibrosarcoma	1.35E-7	–6.913	–2.914	20601955
	Myxoid/round cell liposarcoma	1.53E-5	–5.634	–2.140	20601955

The expression of HK2 mRNA in human tumor samples was further analyzed by TIMER database. Figure 1B shows the differentially expression of HK2 in different tumor tissue samples and normal tissue samples. Compared with normal tissue samples, the expression level of HK2 was remarkable increased in BLCA (bladder urothelial carcinoma), CHOL (cholangiocarcinoma), ESCA (esophageal carcinoma), HNSC (head and neck squamous cell carcinoma), KIRC (kidney renal clear cell carcinoma), KIRP (kidney renal papillary cell carcinoma), LUSC (lung squamous cell carcinoma), PRAD (prostate adenocarcinoma), STAD (stomach adenocarcinoma), THCA (thyroid carcinoma) and UCEC (uterine corpus endometrial carcinoma), while it was remarkable decreased in COAD (colon adenocarcinoma), KICH (kidney chromophobe), LUAD (lung adenocarcinoma) and READ (rectum adenocarcinoma).

### Transcriptional Levels of Hexokinase 2 in Esophageal Carcinoma Patients

The ESCA cohort of TCGA and GEO was utilized to analyze the differential expression of HK2 in ESCA tissue samples and normal tissue samples. Both TCGA and GEO cohort analysis indicated that the HK2 expression level in ESCA tissue samples was remarkable higher than that in normal tissue samples (Figures 1C–E). To further verified the accuracy of data investigation, we conducted qRT-PCR and IHC experiments, respectively. The qRT-PCR results indicated that the HK2 mRNA expression in the two ESCA cell lines (ECA109 and KYSE-150) was remarkable higher than that in the normal human esophageal epithelial cells (Het-1A) (Figure 1F). The results IHC staining revealed that HK2 was mainly expressed in the cytoplasm. The HK2 IHC score in tumor tissue samples was remarkable higher than that in paracarcinoma tissue samples (2.32 ± 0.621 vs. 0.84 ± 0.618) (Figures 1G–I, *P* < 0.0001). These results believe that HK2 plays a potential role in the occurrence and development of ESCA.

**FIGURE 2:**
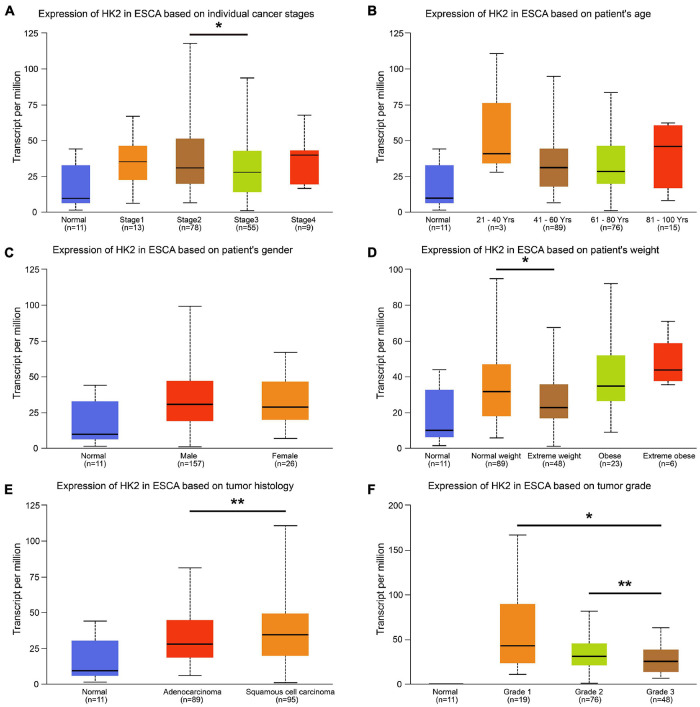
Relationship between HK2 mRNA expression and clinicopathological parameters in esophageal carcinoma (ESCA) patients. The block diagram using the UALCAN network tool shows the correlation between hK2 mRNA expression level and clinicopathological features, including **(A)** individual cancer stages, **(B)** patient age, **(C)** patient gender, **(D)** patient weight, **(E)** tumor histology and **(F)** tumor grade. *P < 0.05; **P < 0.01.

We analyzed clinical data from the ESCA cohort using the UALCAN database to better understand the clinical correlation between HK2 expression and ESCA. The results indicated that the expression of HK2 in cancer stage 2 was higher than that in cancer stage 3 (*P* < 0.05). The expression of HK2 in normal weight patients was higher than that in extreme weight patients (*P* < 0.05). The expression of HK2 in esophageal squamous cell carcinoma was higher than that in esophageal adenocarcinoma (*P* < 0.05). The expression of HK2 in tumor grade 3 was lower than that in tumor grade 2 (*P* < 0.01) and 1 (*P* < 0.05), respectively. However, the expression level of HK2 did not differ in different gender and age groups (Figure 2).

### Enrichment Analysis of Hexokinase 2 Gene Co-expression Network

**FIGURE 3:**
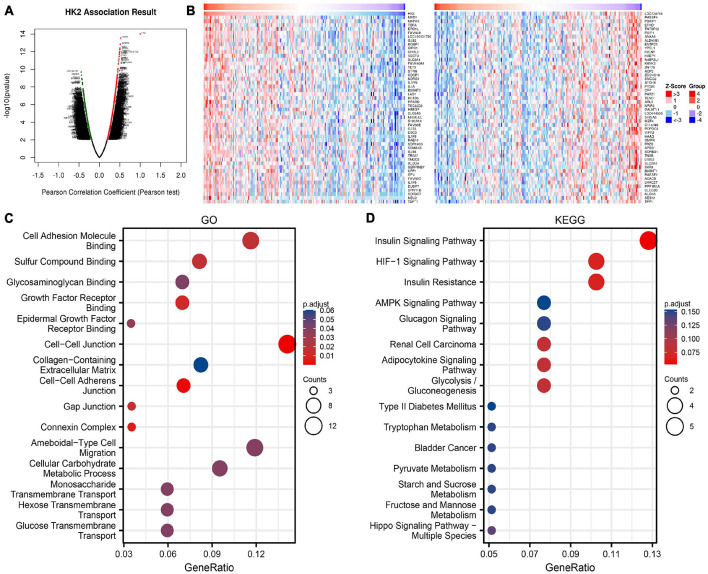
Enrichment analysis of HK2 gene co-expression network in esophageal carcinoma (ESCA). **(A)** The co-expressed genes related to HK2 expression in TCGA ESCA cohort were shown by volcanic map. **(B)** The first 50 co-expressed genes positively and negatively correlated with HK2 expression were shown by heat map. **(C)** Enrichment of gene ontology (GO) terms for HK2 co-expression genes. **(D)** Enrichment of Kyoto Encyclopedia of Genes and Genomes (KEGG) terms for HK2 co-expression genes.

The co-expressed genes related to HK2 expression in TCGA ESCA cohort were analyzed using LinkedOmics database. The analysis results are shown in Figure 3A, 1682 genes were positively associated with the HK2 expression, and 2,855 genes were negatively associated with the HK2 expression (FDR < 0.05). The heat map indicated the top 50 important genes that are positively and negatively associated with HK2 expression, respectively (Figure 3B). Supplementary Table 1 shows all co-expressed genes.

GO function and KEGG pathway research of HK2 co-expressed genes were carried by R language. Under the condition of p.adj < 0.1, HK2 co-expressed genes were participated in 25 biological processes (BP), 6 cell component (CC), 19 molecular function (MF) and 6 KEGG. The bubble graph shows the top 15 messages of GO and KEGG, including 5 messages of BP, CC, and MF. The results of GO functional annotations showed that HK2 co-expressed genes were mainly participated in the cell adhesion molecule binding, cell-cell junction, and ameboidal-type cell migration (Figure 3C). KEGG pathway analysis showed that these genes were mainly related to the insulin signaling pathway, hif-1 signaling pathway, and insulin resistance (Figure 3D). Supplementary Table 2 shows all GO function and KEGG pathway research.

### Analysis of Gene-Gene Interaction and Protein-Protein Interaction of Hexokinase 2 Gene

**FIGURE 4:**
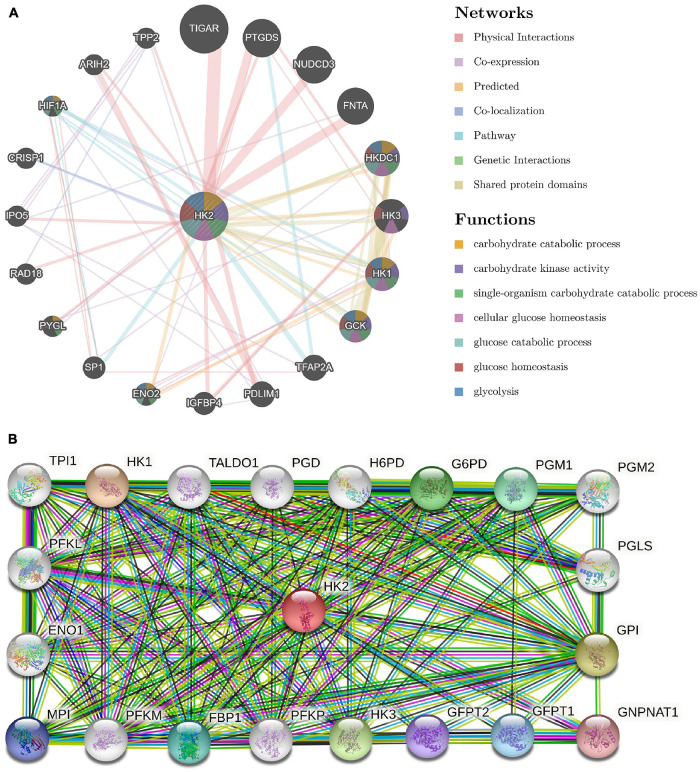
Analysis of gene–gene interaction (GGI) and protein–protein interaction (PPI) of HK2 Gene. **(A)** GGI network of HK2. **(B)** PPI interaction network of HK2.

A GGI network composed of 21 genes was constructed, and the HK2 gene was surrounded by 20 nodes. Their functionality was analyzed using the GeneMANIA database (Figure 4A). These nodes represent genes closely related to HK2 in terms of physical interactions, shared protein domains, predicted, co-localization, pathway, co-expression, and genetic interactions. Analysis showed that there were five genes most related to HK2, which were TIGAR (TP53 induced glycolysis regulatory phosphatase), PTGDS (prostaglandin D2 synthase), NUDCD3 (NudC domain containing 3), FNTA (farnesyltransferase) and HKDC1 (hexokinase domain containing 1). Among them, TIGAR, PTGDS, NUDCD3 and FNTA are related to the physical interaction with HK2, respectively. Both HKDC1 and HK2 have predicted function and shared protein domains. The results also showed that these genes were most strongly associated with glycolysis (FDR = 4.53E-08). In addition, these genes are associated with carbohydrate catabolic process, carbohydrate kinase activity, single-organism carbohydrate catabolic process, cellular glucose homeostasis, glucose catabolic process and glucose homeostasis.

Use STRING to further analyze PPI network of HK2. The results show that HK2 is related to HK1 (hexokinase 1), GPI (glucose-6-phosphate isomerase), HK3 (hexokinase 3), G6PD (glucose-6-phosphate 1-dehydrogenase), PGM1 (phosphoglucomutase-1), FBP1 (fructose-1,6-bisphosphatase 1), GFPT1 (glutamine–fructose-6-phosphate aminotransferase 1), MPI (mannose-6-phosphate isomerase), GFPT2 (glutamine–fructose-6-phosphate aminotransferase 2), GNPNAT1 (glucosamine-phosphate N-acetyltransferase 1), PGM2 (phosphoglucomutase-2), H6PD (hexose-6-phosphate dehydrogenase), PFKM (ATP-dependent 6-phosphofructokinase), TALDO1 (transaldolase), PFKL (ATP-dependent 6-phosphofructokinase), PFKP (ATP-dependent 6-phosphofructokinase), ENO1 (alpha-enolase), TPI1 (triosephosphate isomerase 1) and PGD (6-phosphogluconate dehydrogenase). The combined scores were 0.986, 0.97, 0.967, 0.955, 0.944, 0.941, 0.937, 0.935, 0.933, 0.931, 0.929, 0.925, 0.918, 0.905, 0.866, 0.864, 0.704, 0.66, and 0.495, respectively (Figure 4B).

### Correlation Between Hexokinase 2 and Tumor Immune Infiltrating Cells

**FIGURE 5:**
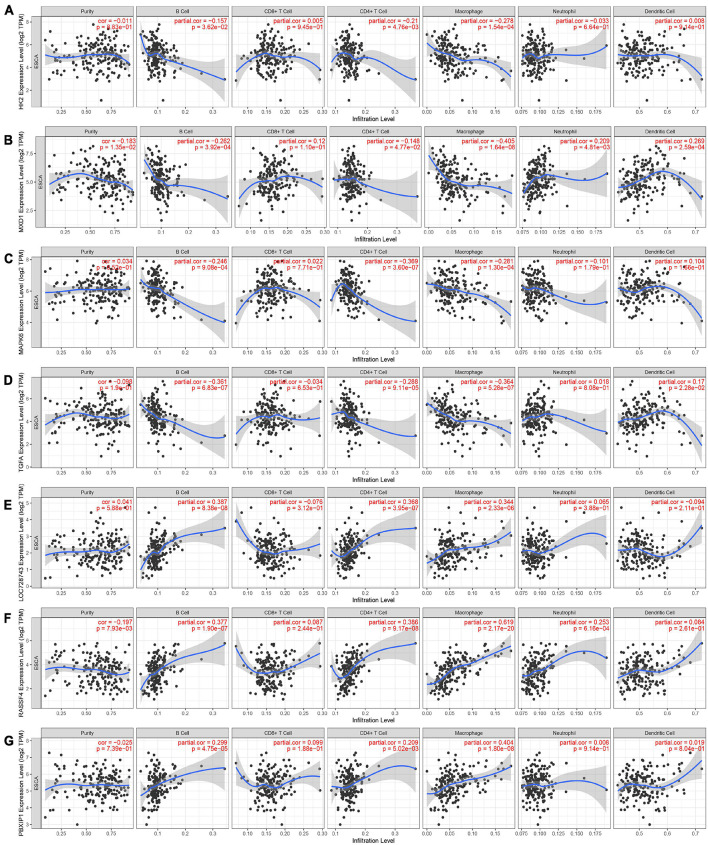
Association between HK2 and tumor immune infiltrating cells. **(A)** Association between the expression of HK2 and the expression of immune infiltrating cell. Correlation between expression of HK2 co-expressed genes and expression in immune infiltrating cells, including MXD1 **(B)**, MAPK6 **(C)**, TGFA **(D)**, LOC728743 **(E)**, RASSF4 **(F)** and PBXIP1 **(G)**.

**FIGURE 6:**
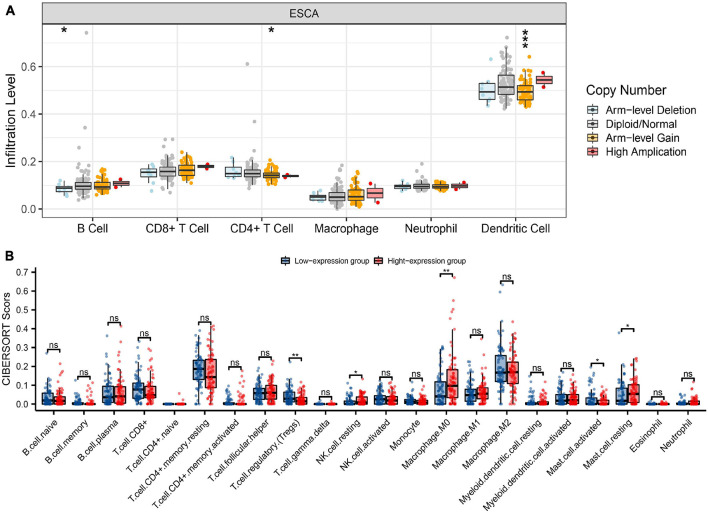
Genetic copy number variations (CNV) of HK2 with the relative abundance of tumor infiltrating cells and CIBERSORT analysis. **(A)** HK2 CNV affects the infiltrating levels of CD4^+^ T cells, neutrophils, and dendritic cells in ESCA. **(B)** The change ratio of 22 immune cell subtypes in the high and low HK2 expression groups in ESCA tumor sample. *P < 0.05; **P < 0.01; ***P < 0.001; ns, no significance.

To explore the relationship between HK2 expression and immune infiltrating cells in ESCA by using TIMER database. Our results indicated that the expression of HK2 was negatively associated with the expression levels of different immune infiltrating cells, including B cell (*r* = –0.157, *P* = 3.62E-2), CD4^+^ T cell (*r* = –0.21, *P* = 4.76E-3) and macrophage (*r* = –0.278, *P* = 1.54E-4) (Figure 5A). In addition, the association between HK2 co-expressed genes and immune infiltrating cells was also analyzed. The first 3 genes positively and negatively correlated with HK2 were selected for correlation analysis of immune infiltrating. We found that HK2 co-expression genes MXD1, MAPK6, TGFA, LOC728743, RASSF4, and PBXIP1 were also significantly associated with the immune cell expression. The expression of MXD1 was associated with B cell (*r* = –0.262, *P* = 3.92E-4), CD4^+^ T cell (*r* = –0.148, *P* = 4.77E-2), macrophage (*r* = –0.405, *P* = 1.64E-8), neutrophil (*r* = 0.209, *P* = 4.81E-3) and dendritic cell (*r* = 0.269, *P* = 2.59E-4) (Figure 5B). The expression of MAPK6 was negatively associated with B cell (*r* = –0.246, *P* = 9.08E-4), CD4^+^ T cell (*r* = –0.369, *P* = 3.60E-7) and macrophage (*r* = –0.281, *P* = 1.30E-4) (Figure 5C). The expression of TGFA was associated with B cell (*r* = –0.361, *P* = 6.83E-7), CD4^+^ T cell (*r* = –0.288, *P* = 9.11E-5), macrophage (*r* = –0.364, *P* = 5.28E-7) and dendritic cell (*r* = 0.17, *P* = 2.28E-2) (Figure 5D). LOC728743 expression was positively associated with B cell (*r* = 0.387, *P* = 8.38E-8), CD4^+^ T cell (*r* = 0.368, *P* = 3.95E-7) and macrophage (*r* = 0.344, *P* = 2.33E-6) (Figure 5E). RASSF4 expression was positively associated with B cell (*r* = 0.377, *P* = 1.90E-7), CD4^+^ T cell (*r* = 0.386, *P* = 9.17E-8), macrophage (*r* = 0.619, *P* = 2.17E-20) and neutrophil (*r* = 0.253, *P* = 6.16E-4) (Figure 5F). PBXIP1 expression was positively associated with B cell (*r* = 0.299, *P* = 4.75E-5), CD4^+^ T cell (*r* = 0.209 *P* = 5.02E-3) and macrophage (*r* = 0.404, *P* = 1.80E-6) (Figure 5G). These results suggest that HK2 and its co-expressed genes may be participated in the immune response of ESCA tumor microenvironment, especially on B cell, CD4^+^ T cell and macrophage. In addition, it was also found that HK2 CNV has a significant correlation with the infiltration level of B cell, CD4^+^ T cell, and dendritic cell (Figure 6A).

CIBERSORT investigation indicated that HK2 expression had associated with tumor immune cell infiltration, including regulatory T cells (*P* = 0.004), resting NK cells (*P* = 0.018), M0 macrophages (*P* = 0.002), activated mast cells (*P* = 0.042) and resting mast cells (*P* = 0.025). Overall, HK2 and its co-expression genes have correlation with tumor immune cell infiltration in ESCA (Figure 6B).

### Correlation Analysis Between Hexokinase 2 Expression and Immune Marker Sets

**FIGURE 7:**
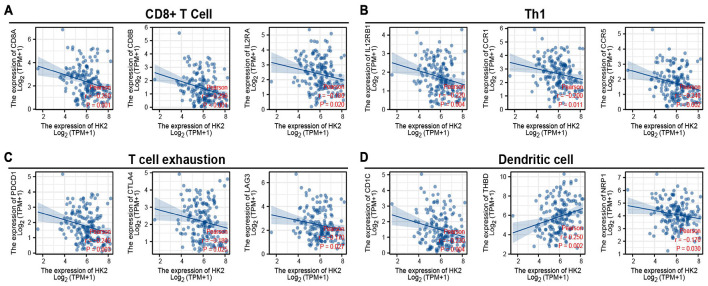
HK2 expression correlated with CD8^+^ T Cell, Th1, T cell exhaustion and dendritic cell in esophageal carcinoma (ESCA). Markers include CD8A, CD8B, and IL2RA of CD8^+^ T Cell **(A)**; IL12RB1, CCR1 and CCR5 of Th1 **(B)**; PDCD1, CTLA4, and LAG3 of T cell exhaustion **(C)**; CD1C, THBD and NRP1 of dendritic cell **(D)**.

To explore the relationship between HK2 and various immune infiltrating cells of ESCA, we used TIMER databases and TCGA ESCA cohort to study the associated between HK2 and immune marker genes of various immune cells (Table 2). Both analyses indicated that the expression of HK2 was associated with the immune marker genes of CD8^+^ T Cell, Th1, T cell exhaustion and dendritic cell. The scatter plots showed the correlation between HK2 expression level and these three immune marker genes, respectively (Figure 7).

**TABLE 2:** Correlation analysis between HK2 and immune cell marker gene in TIMER and TCGA.

**Description**	**Gene markers**	**TIMER**		**TCGA**	
		**Purity**		**Tumor**	
		**Rho**	***P***	**Rho**	***P***
B cell	CD19	–0.108	1.48E-01	–0.144	6.85E-02
	MS4A1	–0.130	8.19E-02	–0.132	9.43E-02
	CD79A	–0.145	5.14E-02	–0.173	**2.73E-02**
CD8^+^ T Cell	CD8A	–0.241	**1.12E-03**	–0.256	**1.03E-03**
	CD8B	–0.258	**4.81E-04**	–0.289	**1.93E-04**
	IL2RA	–0.147	**4.97E-02**	–0.183	**1.98E-02**
Tfh	CXCR3	–0.289	**8.15E-05**	–0.339	**1.03E-05**
	CXCR5	–0.159	**3.26E-02**	–0.145	6.50E-02
	ICOS	–0.115	1.25E-01	–0.149	5.85E-02
Th1	IL12RB1	–0.226	**2.31E-03**	–0.222	**4.48E-03**
	CCR1	–0.152	**4.11E-02**	–0.198	**1.14E-02**
	CCR5	–0.167	**2.53E-02**	–0.240	**2.13E-03**
Th2	CCR4	–0.131	7.94E-02	–0.247	**1.54E-03**
	CCR8	–0.122	1.03E-01	–0.217	**5.46E-03**
	HAVCR1	–0.123	1.01E-01	–0.182	**2.05E-02**
Th17	IL21R	–0.174	**1.96E-02**	–0.248	**1.49E-03**
	IL23R	–0.035	6.42E-01	0.003	9.68E-01
	CCR6	–0.214	**3.84E-03**	–0.196	**1.27E-02**
Treg	FOXP3	–0.147	**4.92E-02**	–0.254	**1.13E-03**
	NT5E	–0.008	9.19E-01	–0.023	7.68E-01
	IL7R	0.029	7.01E-01	–0.018	8.18E-01
T cell exhaustion	PDCD1	–0.251	**6.84E-04**	–0.235	**2.58E-03**
	CTLA4	–0.153	**4.09E-02**	–0.176	**2.49E-02**
	LAG3	–0.155	**3.71E-02**	–0.174	**2.66E-02**
M1 Macrophage	NOS2	–0.022	7.72E-01	0.077	3.30E-01
	IRF5	0.134	7.27E-02	0.165	**3.60E-02**
	PTGS2	0.220	**3.06E-03**	0.229	**3.41E-03**
M2 Macrophage	CD163	–0.143	5.59E-02	–0.181	**2.09E-02**
	MRC1	0.029	7.00E-01	–0.010	9.02E-01
	CD209	–0.150	**4.47E-02**	–0.190	**1.56E-02**
TAM	CCL2	–0.198	**7.84E-03**	–0.209	**7.68E-03**
	CD86	–0.113	1.31E-01	–0.093	2.39E-01
	CD68	0.078	2.97E-01	–0.044	5.81E-01
Monocyte	CD14	–0.167	**2.47E-02**	–0.150	5.70E-02
	CD33	–0.208	**5.11E-03**	–0.207	**8.15E-03**
	ITGAX	0.006	9.40E-01	–0.033	6.77E-01
Natural killer cell	B3GAT1	–0.193	**9.50E-03**	–0.128	1.04E-01
	KIR3DL1	–0.098	1.92E-01	–0.059	4.57E-01
	CD7	–0.289	**8.45E-05**	–0.281	**2.94E-04**
Neutrophil	FCGR3A	–0.103	1.67E-01	–0.166	**3.48E-02**
	CD55	0.028	7.13E-01	0.073	3.58E-01
	ITGAM	–0.020	7.92E-01	–0.104	1.88E-01
Dendritic cell	CD1C	–0.167	**2.53E-02**	–0.227	**3.71E-03**
	THBD	0.224	**2.53E-03**	0.245	**1.67E-03**
	NRP1	–0.070	3.52E-01	–0.171	**3.00E-02**

*Bold values indicate P < 0.05.*

### Hexokinase 2 Expression Is Correlated With m6A RNA Methylation Regulators in Esophageal Carcinoma

**FIGURE 8:**
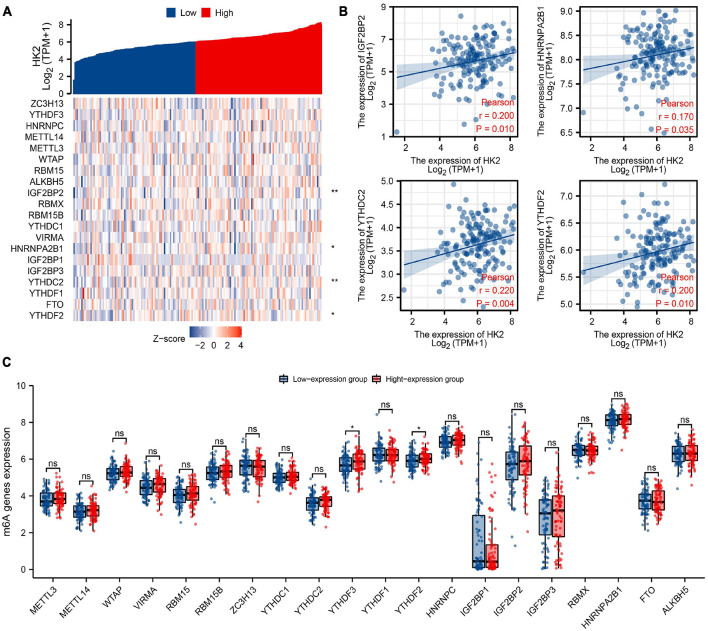
Association of HK2 expression with m6A associated genes in esophageal carcinoma (ESCA). **(A)** The association between the expression level of HK2 and the expression of m6A associated genes in TCGA ESCA cohort was analyzed. **(B)** The association between HK2 and m6A associated genes was displayed by scatter plot, include IGF2BP2, HNRNPA2B1, YTHDC2, and YTHDF2. **(C)** The differential expression of m6A associated genes in the high and low HK2 expression groups was analyzed. *P < 0.05; **P < 0.01; ns, no significance.

The TCGA ESCA cohort was analyzed to study the association between the expression of HK2 and the expression of 20 m6A related genes in ESCA. Analysis indicated that the expression of HK2 was remarkable positively associated with 4 m6A associated genes in ESCA, including IGF2BP2 (*r* = 0.200, *P* = 0.010), HNRNPA2B1 (*r* = 0.170, *P* = 0.035), YTHDC2 (*r* = 0.220, *P* = 0.004) and YTHDF2 (*r* = 0.200, *P* = 0.010) (Figure 8A). The scatter plot indicates the correlation between HK2 and m6A associated genes (Figure 8B). In addition, 162 cases of ESCA were divided into high expression group (*n* = 81) and low expression group (*n* = 81) according to the expression level of HK2. We further evaluated the expression abundance of m6A associated genes between high and low HK2 expression groups to study whether m6A modification was different between the two groups (Figure 8C). Analysis indicated that compared with the low expression group, the expression of YTHDF3 and YTHDF2 were increased in the high expression group of HK2 (*P* < 0.05). These analyses suggest that HK2 is strongly associated to m6A modification in ESCA.

## Discussion

As one of the isozyme subtypes of HK, HK2 is the first rate-limiting enzyme in the catalytic glycolysis pathway and mainly distributed in the cytoplasmic region (Smith, 2000; Zhang et al., 2017). HK2 overexpression can promote the process of glycolysis of tumor cells and provide necessary energy for the proliferation and migration of tumor cells, thus promoting the occurrence and development of tumor cells (Shen et al., 2020; Wang et al., 2020; Yu et al., 2021). Chen et al. (2019) discovered that high expression of HK2 was remarkable associated with the degree of malignancy and poor prognosis of gallbladder tumors. Downregulation of HK2 expression could significantly inhibit the proliferation, migration and invasion of gallbladder cancer cells, and at the same time reduce glucose consumption and cellular lactic acid production. DeWaal et al. (2018) discovered that HK2 knockout inhibited the glycolytic effect of HCC cell, promoted the level of oxidative phosphorylation, and at the same time increased sensitivity of cancer cells to metformin. These results suggest that HK2 can be used as a potential target for cancer gene therapy.

Studies have shown that HK2 is highly expressed in a variety of tumors and plays an important role in the development and progression of tumors. Nevertheless, there are few researches on the comprehensive investigation of HK2 in ESCA. On the project, we predicted the expression differences of HK2 in cancers through bioinformatics investigation, and verified the HK2 expression in ESCA through *in vitro* experiments. Bioinformatics is widely used in tumor research (Chen et al., 2021; Hu et al., 2021; Liao et al., 2021). Investigation of the Oncomine database discovered that HK2 was highly expressed in 8 types of cancer, and investigation of the TIMER discovered that HK2 was highly expressed in 11 types of cancer. According to GEO and TCGA ESCA cohort analysis, the expression level of HK2 in ESCA tissue samples was remarkable higher than that in normal tissue samples. We also detected the expression of HK2 in ESCA sample and normal sample by qRT-PCR and IHC. The investigation results were consistent with the above analysis. At present, we also found that the expression of HK2 was associated to weight, cancer stages, tumor histology and tumor grade. In summary, HK2 may serve as a possible diagnostic and therapeutic biomarker of ESCA.

Nevertheless, previous researches on the role of HK2 in ESCA are limited to the energy metabolism of glycolytic pathway. Liu et al. (2019) found that the stem cell characteristics and abnormal metabolic reprogramming of esophageal cancer stem cells were dependent on the Hsp27-AKT-HK2 pathway, and ESCA patients with overexpression of Hsp27 and HK2 in tumor tissues had the worst prognosis. Mao et al. (2018) found that inhibiting the expression of HK2 could significantly inhibit the glycolytic effect of ESCA and affect the proliferation of cancer cell. Nevertheless, there are few reports on other biological functions of HK2 in ESCA. In this study, the co-expressed genes related to HK2 expression in TCGA ESCA cohort were analyzed using LinkedOmics database. The GO and KEGG function enrichment investigation of 100 co-expressed genes associated to the expression of HK2 indicated that the co-expression of HK2 was significantly correlated to the cell adhesion molecule binding, cell-cell junction, and ameboidal-type cell migration. KEGG pathway investigation indicated that HK2 co-expression was significantly correlated to the insulin signaling pathway, hif-1 signaling pathway, and insulin resistance. All these biological functions and pathways are associated to the occurrence and development of ESCA. GGI investigation discovered 20 genes related to HK2, and these genes were mainly closely related to glycolysis. The three genes most related to HK2 are TIGAR, PTGDS, and NUDCD3, respectively. Studies have shown that inhibition of TIGAR expression can reduce the proliferation of ESCA tumors, and patients who had high expression of TIGAR had poorer prognosis (Chu et al., 2020). PPI analysis showed that HK2 had the strongest correlation with the proteins of HK1, GPI and HK3, and inhibition of the expression of HK1 could significantly inhibit the proliferation of ESCA cells (Li et al., 2014). HK1, HK3, and GPI are all key genes in glycolysis, which can promote the proliferation and development of tumor cells by promoting the glycolysis effect of tumor cells. We discovered that the above three proteins had the highest association with HK2. These researches may suggest an association between HK2 and glycolysis and proliferative development of ESCA. These researches indicated that HK2 is not only involved in the glycolysis of ESCA, but also may play a variety of biological functions in the development and development of ESCA.

Tumor cell infiltration immunity has been shown to be associated with tumor progression and prognosis of ESCA (Baba et al., 2020; Wu X. et al., 2020). We used TIMER database and TCGA cohort to analyze the association between HK2 and ESCA immune infiltrating cells. TIMER database analysis showed that HK2 expression was correlated with B cell, CD4^+^ T cell and macrophage. In addition, the relationship between six co-expressed genes of HK2 and immune infiltrating cells was also analyzed. The co-expressed genes were MXD1, MAPK6, TGFA, LOC728743, RASSF4, and PBXIP1. We found that the expression of these six genes was also closely related to the infiltration of ESCA immune cells, which were all related to the infiltration of B cell, CD4^+^ T cell and macrophage. These results suggest that HK2 and its co-expressed genes may be participated in the immune response of ESCA tumor microenvironment, especially on B cell, CD4^+^ T cell and macrophage. In addition, it was also discovered that HK2 CNV has a remarkable correlation with the infiltration level of B cell, CD4^+^ T cell, and dendritic cell. According to the differential expression of HK2, the proportion of 22 tumor immune cells in ESCA was assessed by CIBERSORT research. We discovered five types of immune cells, including regulatory T cells, resting NK cells, M0 macrophages, activated mast cells and resting mast cell, whose proportions varied significantly according to the expression level of HK2. Furthermore, through the analysis of TIMER database and TCGA ESCA cohort, we discovered that the expression of HK2 was remarkable associated with the gene markers of CD8^+^ T Cell, Th1, T cell exhaustion and dendritic cell. We believe that high expression of HK2 in ESCA patients may trigger an immune response. These results suggest that HK2 plays a remarkable role in the immune regulation of ESCA. Nevertheless, more studies are needed to further certify our speculation.

As part of epigenetics research, m6A is the most common and plentiful modification in post-transcriptional modification of RNA, which can affect the progress of tumor by regulating the biological functions associated with tumor. Shen et al. (2020) found that m6A associated gene METTL3 stabilizes the expression of HK2 and SLC2A1 in colorectal cancer through the m6A-IGF2BP2/3 dependent mechanism, and enhances the glycolysis ability of tumor cells, thus promoting the progress of colorectal cancer. Wang et al. (2020) discovered that METTL3 enhanced the stability of HK2 through YTHDF1-mediated m6A modification, thus promoting the Warburg effect of cervical cancer, and finally promoting the malignant proliferation of tumors. On the project, we attempted to analyze whether the expression level of HK2 is associated to the m6A modification in ESCA. We discovered that the expression of HK2 was remarkable associated with IGF2BP2, HNRNPA2B1, YTHDC2, and YTHDF2. We also discovered that the expression levels of YTHDF3 and YTHDF2 were remarkable increased in the group with high expression of HK2. We believe that HK2 gene may have a regulatory relationship with m6A modification, and this regulatory mechanism is involved in glycolysis and malignant proliferation of ESCA.

## Conclusion

In conclusion, we verified that HK2 is overexpressed in ESCA, and its expression level is associated to clinical case characteristics. The expression level of HK2 is strongly associated to the degree of immune cell infiltration. HK2 is associated with m6A modification, which may enhance the stability of HK2 through m6A modification, and thus promote the glycolytic effect and malignant proliferation of ESCA. HK2 can be used as a potential biological target for diagnosis and therapy of ESCA.

## Data Availability Statement

The datasets presented in this study can be found in online repositories. The names of the repository/repositories and accession number(s) can be found in the article/Supplementary Material.

## Ethics Statement

The studies involving human participants were reviewed and approved by the Ethics Committee of Taihe Hospital Affiliated of Hubei University of Medicine. Written informed consent for participation was not required for this study in accordance with the national legislation and the institutional requirements.

## Author Contributions

X-SL conceived the project and wrote the manuscript. X-SL, Y-JC, F-YL, and R-MW participated in data analysis. X-SL, J-ML, FT, D-BZ, WL, YG, and HZ participated in discussion and language editing. Z-JP reviewed the manuscript. All authors contributed to the article and approved the submitted version.

## Conflict of Interest

The authors declare that the research was conducted in the absence of any commercial or financial relationships that could be construed as a potential conflict of interest.

## Publisher’s Note

All claims expressed in this article are solely those of the authors and do not necessarily represent those of their affiliated organizations, or those of the publisher, the editors and the reviewers. Any product that may be evaluated in this article, or claim that may be made by its manufacturer, is not guaranteed or endorsed by the publisher.

## Footnotes

1
www.oncomine.org

2
https://cistrome.shinyapps.io/timer/

3
https://portal.gdc.cancer.gov/

4
www.ncbi.nlm.nih.gov/geo

5
http://ualcan.path.uab.edu/index.html

6
www.linkedomics.org/login.php

7
www.genemania.org

8
www.string-db.org
