# Supplementary material for: Comprehensive Analysis of Hexokinase 2 Immune Infiltrates and m6A Related Genes in Human Esophageal Carcinoma
Source: Front Cell Dev Biol. 2021 Oct 7;9:715883. doi: 10.3389/fcell.2021.715883 (PMC8544599; doi:10.3389/fcell.2021.715883)
Supplement: Supplementary file 3 [file Data_Sheet_1.docx]

Supplementary Material

# Supplementary Methods

## Cell Lines and Cell Culture Reagents

Human ESCA cell lines ECA109 and KYSE-150 and normal human squamous esophageal cell line Het-1A were obtained from the American Type Culture Collection (Manassas, VA, USA). The cells were maintained in DMEM high glucose medium (Hyclone, Logan, UT, USA) supplemented with 10% FBS (Gibco, USA) and 1% antibiotics (penicillin-streptomycin, Gibco, USA).

## RNA Extraction and qRT-PCR

The implementation method refers to previous study. Total RNA was isolated from cells using Trizol reagent (Invitrogen, Carlsbad, CA, USA). Use Prime Script RT reagent kit (Takara, Dalian, China) for reverse transcription, and then use SYBR Prime Script RT PCR kit (Takara, Dalian, China) for qRT-PCR. Use GAPDH as an internal reference and use the 2^-△△Ct^ method to calculate the results. HK2 primer sequences: forward primer CGACAGCATCATTGTTAAGGAG and reverse primer GCAGGAAAGACACATCACATTT. GAPDH primer sequences: forward primer GGAGCGAGATCCCTCCAAAAT and reverse primer GGCTGTTGTCATACTTCTCATGG.

## Immunohistochemistry and analysis

Clinical samples were obtained from 50 patients with ESCA who were surgically treated at Taihe Hospital Affiliated of Hubei University of Medicine from February 2016 to September 2017. The content of HK2 was detected by IHC according to the method previously described. The ESCA tissue and the paracarcinoma tissues were prepared into 3 μm paraffin sections and incubated with mouse monoclonal antibodies of HK2 (1:200, Abcam, USA) at 4℃ overnight in a refrigerator. The sections were coupled with the goat anti-mouse IgG-HRP secondary antibody (1:2000, Abcam, USA) at room temperature for 1.5 h, then each incubated section was stained with DAB reagent, and finally counterstained with hematoxylin.

IHC staining scores of HK2 were assessed by two experienced observers. IHC score of tumor cells was 0-3: 0, negative; 1, weak; 2, medium; 3, strong.

# Supplementary Tables

## Supplementary Table 1. HK2 co-expressed genes

## Supplementary Table 2. The GO and KEGG enrichment analysis of HK2 co-expression genes
